# Neurophysiological Effects of a Singing Bowl Massage

**DOI:** 10.3390/medicina58050594

**Published:** 2022-04-26

**Authors:** Nike Walter, Thilo Hinterberger

**Affiliations:** Section of Applied Consciousness Sciences, Department for Psychosomatic Medicine, University Hospital Regensburg, 93053 Regensburg, Germany

**Keywords:** mind–body intervention, complementary therapy, singing bowl, EEG, HRV

## Abstract

*Background and Objectives:* In recent years, singing bowl sound interventions have been progressively implemented in the fields of well-being, therapy and education; however, the effectiveness has only scarcely been investigated. Therefore, this study was aimed at determining neurophysiological effects of a singing bowl massage. *Materials and Methods:* In this prospective cohort study 64-channel EEG, ECG and respiration was recorded from 34 participants (mean age 36.03 ± 13.43 years, 24 females/10 males) before, during and after a professional singing bowl massage. Further, subjective changes in well-being were assessed. EEG data were analyzed by determining the effect sizes of distinct frequency bands. Significant differences were calculated by a two-tailed *t*-test corrected for multiple comparisons. Heart rate variability metrics, heart rate and respiration rate were estimated and compared. *Results:* Overall EEG power decreased during the sound condition compared to a task-free resting state (d = −0.30, *p* = 0.002). After the intervention, global EEG power was further reduced (d = −0.46, *p* < 0.001), revealing a decrease in the beta 2 (d = −0.15, *p* = 0.002) and the gamma frequency band (d = −0.21, *p* = 0.004). The mean heart rate was significantly lower after the intervention (75.5 ± 19.8 vs. 71.5 ± 17.9, *p* < 0.001) and the respiration rate higher (13.5 ± 5.3 vs. 15.2 ± 6.3, *p* = 0.018). 91.2% of the participants felt more integrated, 97.1% more balanced and 76.5% more vitalized. *Conclusions*: The neurophysiological effects of a singing bowl sound massage may be interpreted as a shift towards a more mindful, meditative state of consciousness. The intervention was perceived as beneficial for the wellbeing.

## 1. Introduction

Since centuries ago, the concept of sound healing has been utilized by various cultures all over the world. During the last few years, music-based interventions have been progressively implemented in western health care [1]. Such nonpharmacological therapy has been shown to alleviate a variety of symptoms including pain, anxiety and stress, as well as to improve quality of life in pediatrics [2]. In addition, for adults, beneficial outcomes of music therapy were reported, amongst others, in the context of depression [3,4,5] and neurological disorders [6,7,8]. With increasing evidence for the therapeutical effects of the mindfulness concept [9,10], traditional techniques using acoustic stimuli for the induction of meditative states, such as singing bowl sound, were further investigated [11]. Thus, some studies provided first insights into the physiological and psychological effects of singing bowl sound, comparing pre- and posttreatment parameters by analysis of variance [12,13]. However, whereas existing studies applied heart rate variability (HRV) measures and psychometric data, neurophysiological effects of singing bowl applications are unknown. Further, most of the studies chose a study design using listening to the sound of singing bowls as an intervention. In the meantime, the application of directly placing singing bowls on the patient’s body, termed sound massage, has found its way in various applications in the fields of well-being, therapy and education [14]. Therefore, this present study aimed to investigate, for the first time, neurophysiological effects of a singing bowl sound massage. For this purpose, electrophysiological correlates were identified during the intervention using 64-channel EEG and compared to a task-free resting state. Further, it aimed to determine changes in HRV and respiration rate, as well as subjective changes in wellbeing, associated with the singing bowl sound massage intervention.

## 2. Materials and Methods

### 2.1. Data Acquisition and Participants

**Figure 1:**
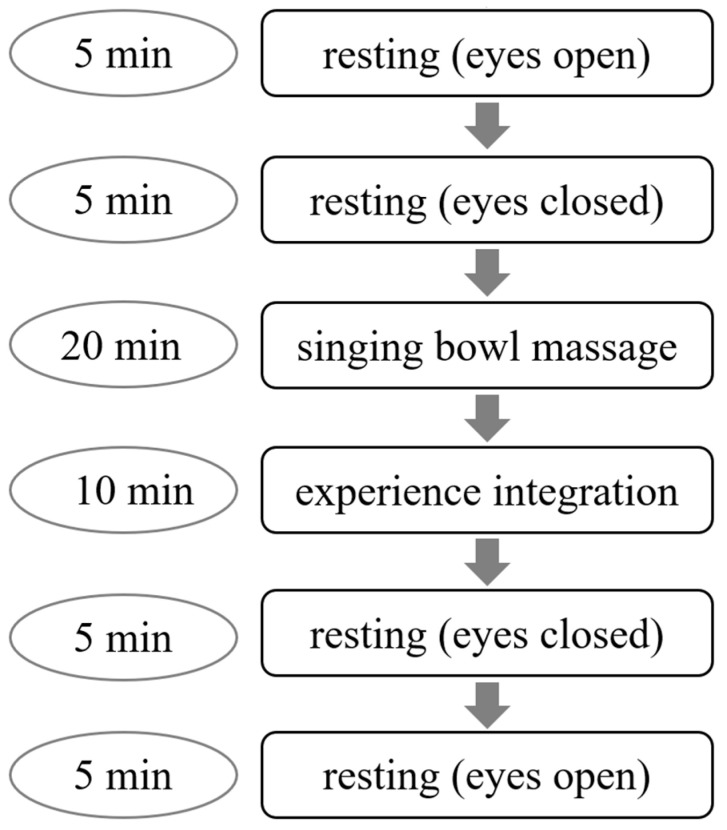
Schematic overview of the experimental procedure.

In this prospective cohort study, electrophysiological data were recorded from 34 participants (mean age 36.03 ± 13.43 years, 24 females/10 males). All participants gave their informed consent prior to the study. The study was approved by the institutional ethics committee of the University Clinic Regensburg according to the Helsinki Convention (file number: 20-1995-101). Data were recorded using a 72-channel QuickAmp amplifier system (BrainProducts GmbH, Munich, Germany). EEG was measured with a 64-channel ANT Waveguard electrode cap (ANT B.V., Enschede, The Netherlands) with active shielding and Ag/AgCl electrodes, which were arranged according to the international 10/10 system. In addition, electrocardiograms (ECGs) were measured with two electrodes between the sternum and the left costal arch. The respiratory signal was recorded from a belt around the lower part of the chest. The experimental procedure started with an initial 10 min baseline resting, including 5 min with eyes open and 5 min with eyes closed. Then, a singing bowl massage was conducted by professionals trained according to the Peter Hess^®^-method, with a duration of 20 min. Afterwards, 10 min of silence were given to integrate the experience. Directly after, a second resting state took place, during which participants kept their eyes closed for 5 min and subsequently opened their eyes for 5 min (Figure 1). During the whole procedure participants lay comfortably on a massage table. Before the recording, the Tellegen-Absorption-Scale (TAS) was measured. This questionnaire captures the absorption capacity (i.e., the individual’s capacity for engaging attentional resources in sensory and imaginative experiences) containing 34 true/false self-report items [15]. After the recording, participants ensued the questionnaire CSP-14, which assesses changes in body sensation, emotional state and mental state. The questionnaire contains 14 items, which are rated on a scale ranging from −3 to +3 [16].

### 2.2. Data Processing

MATLAB (MathWorks, Natrick, MA, USA) was used for data processing. Data were sampled at 250 samples/sec in a range from DC to 70 Hz with a notch filter at 50 Hz. After detrending the 64 EEG channels, a correction for eye movement was done using a linear correction algorithm [17]. A power spectrum time series was calculated using the Fast Fourier Transform (FFT) for the following frequency bands: delta: 1–3.5 Hz, theta: 4–7.5 Hz, alpha 1: 8–10 Hz, alpha 2: 10.5–12 Hz, beta 1: 12.5–15 Hz, beta 2: 15.5–25 Hz, gamma: 25.5–45 Hz, global: 1–45 Hz. To obtain a measure of the power spectral density (PSD) FFT values were squared and all FFT bins within a frequency band range were averaged. EEG PSD was calculated for each participant, task, electrode, and frequency band.

To determine the effects of the singing bowl massage, the following three phases of the experimental course were compared:

1. sound vs. resting (eyes closed)
2. postresting (eyes closed) vs. sound
3. postresting (eyes closed) vs. resting (eyes closed)

The respiration signal was detrended, downsampled to 25 samples/s and filtered using a third-order Butterworth bandpass filter in the range between 0.002 and 1 Hz. The heart rate (interbeat interval) was calculated by analyzing the R-peaks of the ECG signal. Time domain indices of heart rate variability (HRV), such as standard deviation of NN intervals (SDNN) and root mean square of successive RR interval difference (RMSSD), as well as frequency domain indices of HRV, such as low frequency power (0.04–0.15 Hz) and high frequency power (0.15–0.4 Hz), were quantified.

### 2.3. Statistics

For a comparison of the PSD between the first resting, sound application and second resting, effect sizes of the temporal mean of each frequency band for the respective phase were calculated, defined as standardized mean differences (Cohen’s d) [18]. Then, effect sizes of all participants were submitted to a paired two-tailed *t*-test calculated across participants and measures. On a level of global field power eight frequency bands and three comparisons were considered, resulting in 24 variables. These were corrected for multiple comparison using false discovery rate (FDR) adjustment, which gives the proportion of false discoveries among all discoveries [19]. FDR was applied on all dimensions across task conditions, the eight frequency bands and channels. To estimate correlations between the PSD and the psychometric data, Spearman’s rank correlation was used, after determining that the distribution was not appropriate for parametric testing by the Shapiro-Wilk test. For a comparison between HRV metrics and the respiration rate of the first and the second resting state, a Wilcoxon signed-rank test was applied. Significance was set at *p* < 0.05.

## 3. Results

**Figure 2:**
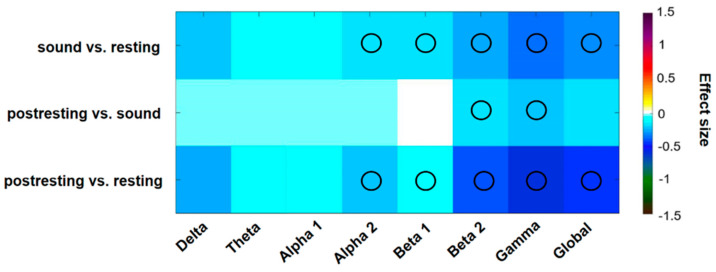
Color-coded differences of power spectral density shown as effect sizes (Cohen’s d) of the task comparisons. Fields marked with a black circle were significant on a 0.05 level after FDR adjustment.

For a detailed comparison between the three phases, effect sizes were estimated for each condition and frequency band. Significant differences were determined by a two-tailed *t*-test corrected for multiple comparisons by the false discovery rate. Comparing the distinct phases of the course of experimental procedure, there was significantly less overall EEG power during the sound condition compared to the first resting state (d = −0.30, *p* = 0.002). The decrease of EEG activity was specifically significant for the frequency bands alpha 2 (d = −0.17, *p* = 0.003), beta 1 (d = −0.16, *p* = 0.002), beta 2 (d = −0.24, *p* = 0.005), and gamma (d = −0.35, *p* = 0.001). The comparison between the second resting state and the sound condition revealed a decrease in the beta 2 (d = −0.15, *p* = 0.002) and the gamma frequency band (d = −0.06, *p* = 0.004). Further reduction in global EEG power was observed during the second resting states compared to the first resting state (d = −0.46, *p* < 0.001), also with significant effects for alpha 2 (d = −0.21, *p* = 0.010), beta 1 (d = −0.14, *p* = 0.006), beta 2 (d = −0.40, *p* < 0.001) and gamma (d = −0.21 *p* < 0.001) (Figure 2).

There was no statistically significant change regarding HRV metrics. The mean heart rate was significantly lower after the intervention (75.5 ± 19.8 vs. 71.5 ± 17.9, *p* < 0.001) and the respiration rate was higher (13.5 ± 5.3 vs. 15.2 ± 6.3, *p* = 0.018) (Table 1).

Regarding the subjective effects of the singing bowl massage, 91.2% of the participants felt more integrated, 97.1% more balanced and 76.5% more vitalized. The bodily feeling was rated as wider (85.3%), more intense (91.2%), more relaxed (91.2%), more comfortable (88.2%) and more powerful (70.6%). The emotional state appeared to be calmer for 82.4% of the participants. Further, after the singing bowl application, participants reported to be happier (79.4%), satisfied (88.2%), more secure (82.4%) and connected (88.2%). Mentally, the majority of participants felt clearer (73.5%). Regarding the phenomenology of consciousness, participants scored highest in the dimensions of “openness”, “memory”, “introversion”, “timelessness”, “imagination” and “cognitive clarity”. The mean TAS score was 69.4 ± 27.5. The total TAS scores did not correlate significantly with age (*Spearman’s* ρ = 0.19, *p =* 0.271) or sex (*Spearman’s* ρ = −0.144, *p =* 0.417). The highest correlation was found between the gamma band power during the first resting state and the total TAS score, however, it was not statistically significant (*Spearman’s* ρ = 0.299, *p =* 0.097). The dimensions of the CSP-14 did not significantly correlate with the PSD in any frequency band during the three experimental phases, respectively.

## 4. Discussion

The intervention of a singing bowl massage found its way into various fields of application such as prevention, therapy, wellness and education. In this study, neurophysiological effects as well as subjective changes of wellbeing were evaluated. The results showed an overall decrease of EEG power during the singing bowl massage, as well as afterwards. The effects were most pronounced in the beta 2 and gamma frequency band.

The neurophysiological changes may be interpreted as a refrain from specific cognitive processing such as mental conceptualization, which would be commensurate with the essential aspect of mindfulness, namely non-judgmental awareness of the moment-to-moment-experience [20]. This would be in line with another study by Hinterberger and colleagues, reporting global decreased EEG activity as well as decreases in the frontal beta and the central and parietal gamma band, when highly experienced meditators entered a state of thoughtless emptiness [21]. Further, a decrease in power over all frequency bands was detected during a meditation characterized as “sacred, unified, egoless, and blessed” [22]. In addition, Dor-Ziderman et al. distinguished between a state of “narrative” self-awareness and “minimal” self-awareness in a MEG neurophenomenological study. The authors reported that the first involved frontal and medial prefrontal gamma band power decrease, while the latter was related to a beta band power decrease in a network, including ventral medial prefrontal, medial posterior and lateral parietal regions. Furthermore, the authors linked an attenuation of beta band activity in the right inferior parietal lobule to a state of selflessness [23]. The reported positive psychological effects are in line with other findings in the literature. For instance, Goldsby et al. reported less tension, anger, fatigue, and depressed mood (*p* < *0*.001) after a meditation with Tibetan singing bowl in healthy participants, and the feeling of spiritual well-being was significantly higher [13]. In addition, an improvement in positive affect and a reduction in negative affect, as captured by the Positive and Negative Affect Schedule (PANAS) questionnaire [24], was reported after a 40-min-long sound meditation with singing bowls [25]. In the presented study, participants also reported to feel more vitalized, which is in accordance with another study determining that subjective sleepiness was lower after a 20 min relaxation session with singing bowl sound compared to a silent relaxation (*p* = 0.041) [26]. Further, listening to singing bowl sound was shown to be a useful strategy to reduce anxiety in patients waiting for urologic surgery [27], which is consistent with high percentages of participants reporting to be more relaxed, calm and balanced in this study.

In line with the findings of a significantly decreased heart rate, Landry compared the effects of a directed relaxation session with and without the use of Himalayan singing bowl sound in a randomized controlled trial, reporting a decline in systolic blood pressure (*p* = 0.044) and heart rate (*p* = 0.003) in the first group [12]. Whereas here no significant changes were found in HRV metrics, a significant increase in heart rate variability was observed when applying singing bowl sound during a relaxation session compared to silent relaxation [28].

Here, no statistically significant correlation between the TAS scores and the frequency band power was found. However, other findings suggested that absorption, as a personality characteristic may be a predictor of outcomes in mind–body interventions [29]. For instance, fibromyalgia patients with high levels of absorption reported more clinically relevant improvements after a guided imagery intervention in a randomized, controlled trial [30].

The presented findings suggest that the application of a singing bowl massage is beneficial on a physical and psychological level. Addressing the question whether the effects are also therapeutical, Wepner and colleagues investigated singing bowl massage interventions in patients with chronic unspecific pain. In their study, participants were divided into three groups, either receiving singing bowl therapy, a placebo intervention, or no treatment; both the placebo and the treatment group showed less pain intensities [31]. However, in a recent review it was concluded that more evidence is required to recommend singing bowl therapies as numbers of studies eligible for inclusion were small (*n* = 4) [11].

This study shows several limitations. First, the sample size may limit the findings in generalizability. In addition, this study was conducted solely with healthy participants and for the translation into clinical practice, more evidence based on randomized controlled trials is required. Further, the measurement and laboratory setting may have influence subjective changes in wellbeing. In addition, no comparison with other interventions such as relaxation without sound, or solely listening to singing bowl sound, were made and thus, blinding of participants was not feasible. However, strengths of the study include the novelty of identifying singing bowl massage effects on a psychological, physiological and neurological level for the first time.

## 5. Conclusions

In conclusion, neurophysiological correlates of a singing bowl sound massage were identified. These were characterized by a decrease of overall EEG power most pronounced in the beta 2 and gamma frequency band, which might reflect a mindful state of consciousness. Subjectively, the intervention was perceived as beneficial for the wellbeing.

## Figures and Tables

**Table 1:** Heart rate variability metrics and respiration rate for each condition.

Parameter	Resting [Mean (SD)]	Sound [Mean (SD)]	Postresting [Mean (SD)]	Comparison (*p*-Value)
HR [bmp]	75.5 (19.8)	71.3 (17.0)	71.5 (17.9)	<0.001 *
SDNN [ms]	68.5 (36.0)	68.3 (34.5)	73.0 (43.3)	0.152
RMSSD [ms]	52.4 (34.6)	54.5 (34.6)	55.7 (38.7)	0.057
LF power [%]	41.6 (19.6)	36.7 (14.1)	39.0 (18.0)	0.203
HF power [%]	21.8 (13.1)	25.0 (14.1)	21.9 (13.2)	0.717
Respiration rate	13.5 (5.3)	14.0 (6.5)	15.2 (6.3)	0.018 *

SDNN = Standard deviation of NN intervals, RMSSD = root mean square of successive RR interval differences, HR = heart rate, LF = low frequency, HF = high frequency. * *p* < 0.05.

## Data Availability

The datasets generated and analyzed in the current study are available from the corresponding author on reasonable request.
